# The impact of Panx1 on inflammation, immunity, and cancer: a comprehensive review

**DOI:** 10.3389/fmed.2025.1572418

**Published:** 2025-09-04

**Authors:** Francisco J. Bravo, Javier Mena, Ángel Mejía Reyes, Carolina Schäfer, Nelly Núñez-Rojas, Cristopher Blamey-Fredes, Claudio Acuña-Castillo, Carlos Barrera-Avalos

**Affiliations:** ^1^Centro de Biotecnología Acuícola, Facultad de Química y Biología, Universidad de Santiago de Chile, Santiago, Chile; ^2^Departamento de Biología, Facultad de Química y Biología, Universidad de Santiago de Chile, Santiago, Chile

**Keywords:** Pannexin-1 (Panx1), purinergic signaling, inflammation and immunity, tumor microenvironment, Panx1-based therapies

## Abstract

Pannexin 1 (Panx1) is a widely expressed membrane channel that regulates ATP release and purinergic signaling, playing essential roles in inflammation, immunity, and tumor progression. This review provides a comprehensive analysis of its structure, activation mechanisms, and its functional relevance in both innate and adaptive immunity. Panx1 has been implicated in inflammasome activation, neutrophil and dendritic cell regulation, and modulation of immune responses against infections, including SARS-CoV-2. Additionally, Panx1 plays a dual role in tumor progression, acting either as a promoter or a suppressor depending on the cellular and microenvironmental context. Pharmacological inhibition of Panx1 has shown therapeutic benefits in preclinical models of inflammatory, cardiovascular, and neurodegenerative diseases, establishing it as a promising and versatile therapeutic target. This review underscores the need for further research into Panx1’s molecular mechanisms and the development of targeted interventions to effectively address inflammatory and autoimmune diseases with precision and efficacy.

## 1 Introduction

Cells communicate with each other through various mechanisms, including the release of signaling molecules into the extracellular space. These intercellular communication systems are essential for coordinating tissue and organ function in multicellular organisms (1). In 2000, a novel family of transmembrane proteins named pannexins was identified by Panchina et al. (2), based on their sequence homology to invertebrate innexins, although they are structurally distinct from vertebrate connexins. Pannexins form channels that facilitate the passage of ions and small signaling molecules across the plasma membrane, contributing to paracrine and autocrine communication. Among the three pannexin isoforms, Pannexin 1 (Panx1) is the most widely expressed and functionally characterized. It has emerged as a key mediator in several physiological and pathological contexts, including inflammation, immune regulation, and tumor progression (3). Panx1 is found in a broad range of cell types, including immune cells, where it regulates the release of ATP and other danger-associated molecular patterns (DAMPs) that modulate inflammation and immune activation (4). The clinical and biological relevance of Panx1 is increasingly evident. Its dysregulation has been associated with various inflammatory pathologies, such as sepsis, rheumatoid arthritis, and inflammatory bowel diseases, as well as with cancer development and progression (5). Therefore, understanding the role of Panx1 in immune modulation is crucial for developing new therapeutic strategies for these diseases.

This review aims to consolidate the most relevant and up-to-date information on Panx1, exploring its structure, regulation, and functions in innate and adaptive immunity, inflammation, and the tumor microenvironment. Additionally, it examines the therapeutic perspectives related to Panx1 modulation, highlighting its significance in inflammatory diseases and cancer.

## 2 Structure and function of Panx1

Pannexin 1 (Panx1) is a membrane protein that assembles into channels composed of seven identical subunits (heptamers) (6). Pannexins were first described in 2000 by Panchina et al. (2), who identified a novel family of proteins homologous to invertebrate innexins but distinct from vertebrate connexins. Among them, Pannexin 1 (Panx1) was the most ubiquitously expressed member. Later, in 2003, Bruzzone et al. (7) demonstrated that Panx1 could form functional channels when expressed in Xenopus oocytes, marking a key step in its functional characterization.

**Figure 1:**
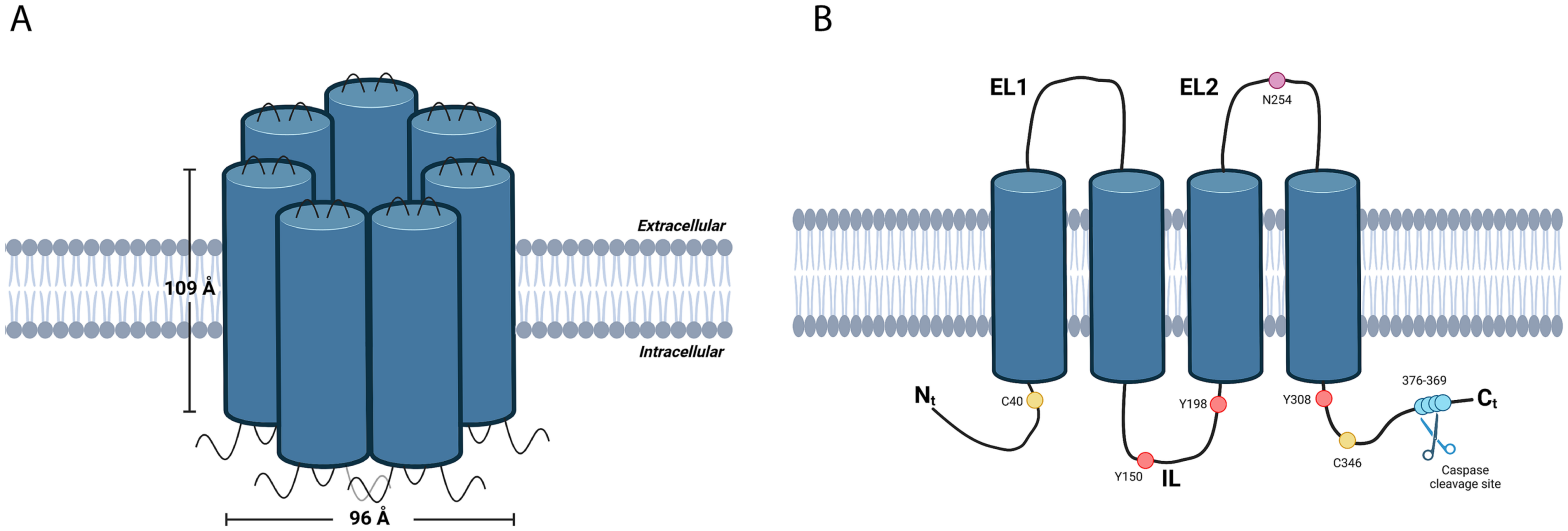
**(A)** Schematic representation of Pannexin 1 (Panx1), a membrane protein from the connexin family that assembles into homoheptamers to form hemichannels. Each subunit spans the membrane through four domains, with both the N-terminal and C-terminal ends oriented toward the cytoplasm. The complex forms a central pore that facilitates the passage of ions and small molecules, while the extracellular region interacts with ligands and other proteins. **(B)** Diagram of a Panx1 subunit highlighting the main post-translational modification sites that regulate channel opening and closing mechanisms. The image highlights glycosylation (pink dots), which controls cellular trafficking; caspase-mediated C-terminal cleavage (blue dots), which permanently activates the channel; S-nitrosylation (yellow dots), which inhibits channel activity; and phosphorylation (red dots), which modulates its opening and permeability. Created with BioRender.com.

Studies in *Xenopus laevis* oocytes expressing Panx1 have demonstrated that its three-dimensional structure, determined through cryo-electron microscopy (cryo-EM), has revealed important details about its architecture and function (8, 9). The channel features a central pore that allows the passage of ions and small molecules, as well as an extracellular region that interacts with ligands and other proteins. Both the N-terminal and C-terminal ends of Panx1 are located in the cytoplasm and play crucial roles in channel regulation. The C-terminal end can block the channel pore and inhibit its opening (10) (Figure 1A).

The structural analysis has also provided insights into the channel’s opening and closing mechanisms, which are regulated by various factors, including changes in membrane potential, ligand binding, and post-translational modifications. For instance, its function is modulated by post-translational modifications at key sites, such as glycosylation, which controls its cellular trafficking; caspase cleavage (R376-379), which permanently activates the channel; S-nitrosylation at C40 and C346, which inhibits it; and phosphorylation at Y150, Y198, and Y308, which regulates its opening and permeability (11) (Figure 1B).

### 2.1 Other pannexin isoforms and their relevance

While Pannexin 1 (PANX1) is the most widely studied member of the pannexin family due to its broad expression and functional involvement in inflammation, immunity, and cancer, the other isoforms—PANX2 and PANX3—also exhibit tissue-specific expression and may play context-dependent roles in physiology and disease. Pannexin 2 (PANX2) was initially described as being restricted to the central nervous system; however, more recent studies have revealed a broader expression profile, including the skin, where it is primarily localized in the suprabasal layers of the epidermis and in dermal fibroblasts (12). In epidermal keratinocytes, PANX2 expression appears to be regulated during cellular differentiation and has been associated with increased susceptibility to UVB-induced apoptosis, suggesting a potential role in skin homeostasis and in local immune responses mediated by programmed cell death. Furthermore, a recent transcriptomic and bioinformatic analysis identified PANX2 as a potential immunological biomarker in gliomas, where its expression negatively correlates with immune cell infiltration, implying a context-dependent immunomodulatory role (13). Pannexin 3 (PANX3) is predominantly expressed in musculoskeletal tissues such as bone, cartilage, and skin, where it plays essential roles in regulating cell differentiation, particularly in osteoblasts, chondrocytes, and keratinocytes. Its expression is tightly regulated during osteogenesis and chondrogenesis, acting as an endoplasmic reticulum Ca^2+^ channel, a plasma membrane hemichannel, and as a component of gap junctions, thereby facilitating intercellular communication critical for cellular maturation (14). In the skin, PANX3 also modulates local inflammatory responses and wound healing, as observed in Panx3-deficient mouse models, which exhibit delayed skin repair (15). While its role in systemic immunity remains to be fully elucidated, these findings suggest a potentially relevant immunomodulatory function in localized tissue inflammation.

### 2.2 Channel activation and regulation mechanisms

To date, several activation mechanisms have been described, involving electrical and chemical stimuli, as well as post-translational modifications of the protein. Membrane depolarization is a physiological process that occurs under both homeostatic and pathological conditions. In the case of PANX1, sustained or abnormal depolarization can lead to channel opening, contributing to ATP release and downstream signaling (7, 16–18). Although the molecular processes underlying its activation are not yet fully understood, an interaction between N-methyl-D-aspartate (NMDA) receptors and Panx1 channels has been observed under excitotoxic conditions, and it has been suggested that phosphorylation of its C-terminal end by Src kinase may mediate anoxia-induced activation (19). Additionally, an increase in extracellular potassium associated with neuronal hyperactivity can also activate Panx1 independently of depolarization (20).

As previously demonstrated by Locovei et al. (21) Panx1 channels are activated by both extracellular ATP, through purinergic P2Y1 and P2Y2 receptors, and cytoplasmic calcium at micromolar concentrations, playing a key role in the initiation and propagation of intercellular calcium waves. These waves, essential for cellular communication, propagate regeneratively via ATP release and the activation of P2Y receptors, with distinct activation kinetics: slow and sustained in P2Y1, and rapid and transient in P2Y2. Using *Xenopus laevis* oocytes and patch-clamp techniques, it was confirmed that Panx1 functions as a specialized ATP-release channel in response to stimuli such as mechanical stress, depolarization, or calcium increases, distinguishing itself from other evaluated channels.

The activation of Panx1 channels by caspases occurs through a mechanism in which caspase-3 and caspase-7 cleave the channel’s C-terminal region, removing its autoinhibitory segment. This structural cleavage “unlocks” the channel, leading to its irreversible opening and allowing the uncontrolled release of ATP and other small metabolites into the extracellular space (22). This activation is critical in the context of inflammation and apoptosis, as the released ATP acts as a danger signal, stimulating purinergic receptors such as P2X7, which in turn enhances inflammation by triggering inflammasome activation (23).

Yang et al. (24) reviewed the mechanical stimulation mechanisms of the Panx1 channel, highlighting that its activation can occur in response to various mechanical forces applied to the cell membrane. It was described that Panx1 can open through the “force-from-lipids” model, in which tension in the lipid bilayer induces channel opening, or through the “force-from-filaments” model, which involves interactions with the cytoskeleton or extracellular matrix. Previous studies have shown that mechanical stress, such as hydrostatic pressure, cell swelling, or mechanical deformation, activates Panx1 in various cell types, including erythrocytes, pulmonary epithelium, and neurons, promoting ATP release [reviewed in Yang et al. (24)]. These findings underscore the relevance of Panx1 as a key mechanosensitive channel and its potential impact on intercellular communication as well as physiological and pathological processes.

Although ATP is the most extensively studied molecule released through Pannexin 1 (Panx1) channels, other small metabolites have also been identified. Lactate has been identified as another key metabolite exported via Panx1 in CD8+ T cells. Vardam-Kaur et al. (25) demonstrated that Panx1-mediated lactate efflux supports effector T cell differentiation and memory formation by promoting mitochondrial respiration and AMPK activation. Lastly, during SARS-CoV-2 infection, prostaglandin E2 (PGE2) was shown to be released via Panx1 channels, contributing to the cytokine storm and pulmonary inflammation observed in COVID-19 patients (26). Together, these findings underscore that Panx1 channels serve as conduits for a broader range of signaling molecules beyond ATP, which can amplify inflammation, modulate immune responses, and influence disease progression in various pathological contexts.

Taken together, the wide variety of Panx1 activation mechanisms—including electrical, chemical, proteolytic, and mechanical stimuli—highlights the versatility of this channel as a sensor of cellular stress. Despite significant advances, the precise molecular pathways regulating its activation under physiological versus pathological conditions remain incompletely defined. Future research should focus on integrating these mechanisms in tissue-specific contexts and determining how the type and intensity of stimulus modulate downstream signaling. Clarifying these aspects may be key to targeting Panx1 therapeutically in inflammation, neurodegeneration, or cancer.

### 2.3 Post-translational modifications and protein interactions

Post-translational modifications play a crucial role in regulating Panx1 function. Glycosylation regulates Panx1 localization and its interaction with other proteins (3, 27). Specifically, N-glycosylation promotes Panx1 trafficking to the plasma membrane, enabling its functional expression at the cell surface (28). Moreover, phosphorylation of Panx1 by protein kinase C (PKC) facilitates channel opening, thereby enhancing its activity in response to extracellular stimuli (29).

Moreover, protein interactions also influence Panx1 function. Using Panx1-deficient mouse models and J774 macrophage cell lines, it has been shown that Panx1 interacts with the purinergic receptor P2X7, and this interaction can amplify ATP release and purinergic signaling (30). Furthermore, long-term Panx1 deletion in CA1 hippocampal pyramidal neurons led to significant structural and functional modifications. Panx1-KO neurons exhibited increased excitability, dendritic complexity, and spine maturation compared to wild-type animals, accompanied by an increase in postsynaptic density and multiple synaptic contacts. These alterations were associated with changes in actin cytoskeleton dynamics, including increased polymerization and an imbalance in the activity of the GTPases Rac1 and RhoA. Using electrophysiology, electron microscopy, and Golgi staining, this study highlighted the stabilizing role of Panx1 in synaptic morphology and functionality, mediated by the regulation of actin dynamics (31). Collectively, these activation and regulatory mechanisms ensure that Pannexin 1 functions appropriately in various physiological and pathological contexts, including immune responses.

## 3 Panx1 in immune response

### 3.1 Pannexin 1-mediated release of inflammatory cytokines and chemokines

Panx1 plays a crucial role in regulating the inflammatory response primarily by facilitating the release, rather than the transcriptional induction, of cytokines and chemokines. These signaling molecules coordinate communication among immune cells and promote the progression or resolution of inflammation. Panx1-mediated ATP release serves as a critical danger-associated molecular pattern (DAMP) that activates purinergic P2X7 receptors, leading to inflammasome assembly and the subsequent maturation and secretion of IL-1β (20). In contrast, TNF-*α* is primarily released via conventional vesicular pathways, although Panx1 may indirectly modulate its release through ATP-dependent autocrine signaling. *In vitro* studies using HEK293 cells expressing P2X7 have shown that coactivation of Panx1 and P2X7 receptors amplifies ATP release, further enhancing downstream inflammatory signaling (32).

Panx1 has been implicated in the release of various inflammatory cytokines and chemokines, including IL-1β, TNF-*α*, IL-6, and chemokines such as CCL2 and CXCL1. In murine peritoneal macrophages, Panx1 activation and ATP release induce the activation of the NLRP3 inflammasome, which processes pro-IL-1β into its mature form, IL-1β (32). In colorectal cancer cells, TNF-*α* modulates Panx1 activation to promote ATP release (33). In mice with traumatic brain injury, Panx1 inhibition reduces IL-6 expression in the brain (34). Additionally, a study investigated the role of pannexin-1 (Panx1) in regulating inflammation during necroptosis, a form of programmed cell death. Using HT-29 cells, a human colon adenocarcinoma epithelial cell line, it was demonstrated that MLKL oligomers, activated by RIPK3, induce Panx1 channel opening through the action of the GTPases RAB27A and RAB27B, facilitating selective plasma membrane permeabilization. Although Panx1 was not essential for necroptotic cell death, its inhibition or silencing significantly increased the production of pro-inflammatory cytokines such as IL-8, suggesting that Panx1 acts as an inflammation modulator by limiting the release of these cytokines (35). Interestingly, these findings contrast with previous studies describing Panx1 as an inflammation facilitator, promoting cytokine release—such as IL-1β—during other cellular processes, including apoptosis and pyroptosis (20).

Beyond its canonical role in ATP release and inflammasome activation, Panx1 may also contribute to inflammatory cell death through calcium-dependent mechanisms. In immune cells such as monocytes, macrophages, and neutrophils, Panx1 can promote calcium influx or secondary calcium signaling via purinergic receptors, which may facilitate activation of calpain-1, a calcium-dependent cysteine protease. Recent studies have demonstrated that calpain-1 can cleave Gasdermin D (GSDMD) at a site distinct from the canonical caspase-1 cleavage, suggesting an alternative, caspase-independent pyroptotic pathway (36, 37). The colocalization of calpain-1 with pyroptotic markers in granulocytes and monocytes points to its functional role in regulating immune cell death. Given that excessive or dysregulated pyroptosis can amplify tissue damage in sepsis, neuroinflammation, or inflammatory bowel disease, the potential regulatory axis of Panx1–calcium–calpain-1 could represents a novel mechanism by which immune responses are modulated during inflammation.

This apparent discrepancy may reflect differences in physiological context, dominant signaling pathways, or experimental conditions, highlighting the need for further studies to reconcile Panx1’s dual role in inflammation regulation.

### 3.2 Pannexin 1 in macrophages and monocytes

The role of pannexins has been well established in cells of the innate immune system. For instance, Panx1 channels play a significant role in regulating acute vascular inflammation, particularly by acting as ATP release pathways in venous endothelial cells following TNF-*α* stimulation. Using *in vitro*, ex vivo, and *in vivo* models, it has been demonstrated that Panx1 phosphorylation mediated by Src family kinases (SFK) is crucial for channel opening and subsequent ATP release. This ATP activates purinergic receptors on immune cells, promoting leukocyte adhesion and migration across the venous wall, as previously demonstrated in venous inflammation models (38). Panx1-KO mice exhibited a significant reduction in leukocyte adhesion and migration during acute inflammation, underscoring the essential role of Panx1 in these inflammatory responses. Along the same lines, specific functions of Panx1 have been described in various innate immune cell types, including macrophages, neutrophils, and dendritic cells.

One of the most important roles of Panx1 in macrophages and monocytes is its involvement in inflammasome activation. The inflammasome is a multiprotein complex that activates caspase-1, an enzyme responsible for processing the pro-inflammatory cytokines IL-1β and IL-18 into their active forms (32). Inflammasome activation is a critical process in the innate immune response, and its dysregulation can contribute to the development of chronic inflammatory diseases.

*In vitro* studies using murine peritoneal macrophages have demonstrated that Panx1 activation induces ATP release into the extracellular space (20). Extracellular ATP, in turn, activates the purinergic receptor P2X7, triggering NLRP3 inflammasome activation and IL-1β release. Pharmacological or genetic inhibition of Panx1 reduces this effect, suggesting that Panx1 is a key component in this process.

**Figure 2:**
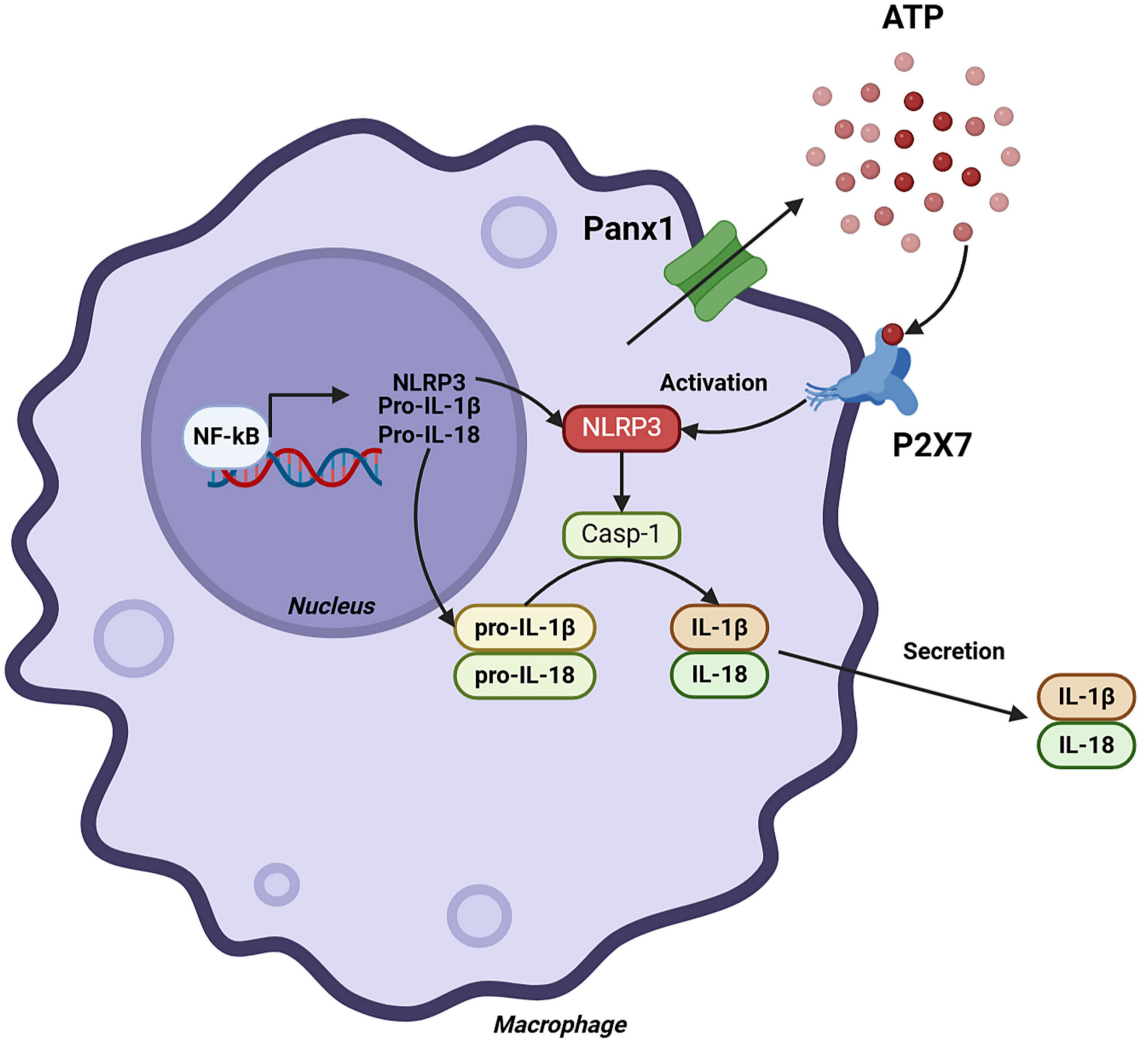
Role of Panx1 in monocytes and macrophages. The figure illustrates the cascade of events leading to the production of IL-1β and IL-18 in macrophages, highlighting the central role of Panx1. Panx1 activation induces ATP release into the extracellular space, which in turn activates the purinergic receptor P2X7. This activation leads to a decrease in intracellular K^+^ concentration, triggering the activation of the NLRP3 inflammasome multiprotein complex. Subsequently, the inflammasome activates caspase-1, which proteolytically processes IL-1β and IL-18 precursors, generating their active forms for secretion. Created with BioRender.com.

**Figure 3:**
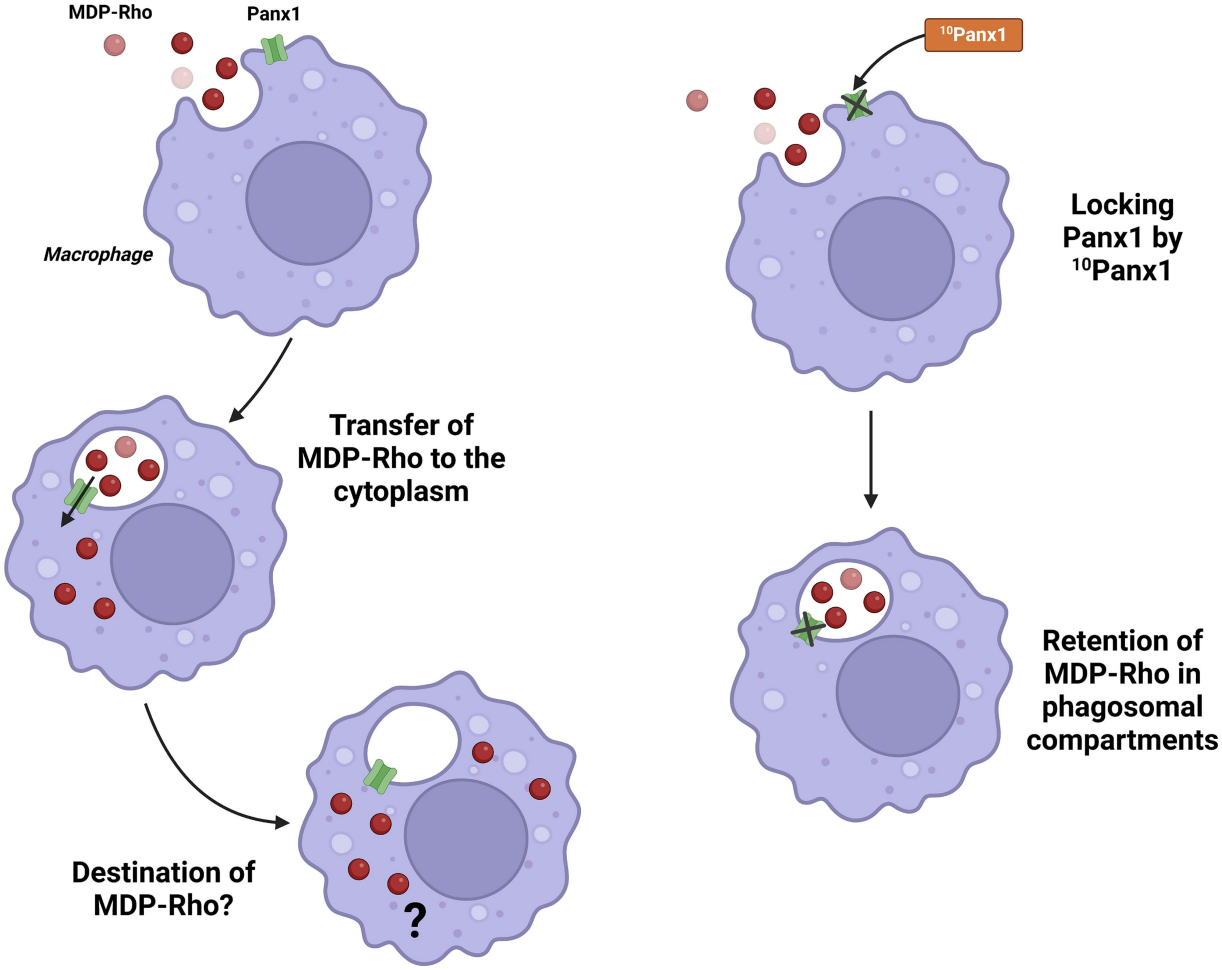
Possible involvement of Panx1 in antigen transfer from phagosomes to the cytoplasm. Panx1 may facilitate the transfer of MDP-Rho into the cytoplasm, potentially linking it to antigen presentation. This effect is inhibited using the ^10^Panx1 inhibitory peptide. Created with BioRender.com.

On the other hand, in macrophages, Panx1 facilitates the activation of the cryopyrin (NLRP3) inflammasome through its interaction with extracellular ATP, acting as an autocrine signal that amplifies inflammation (Figure 2). Studies conducted in primary macrophages and RAW 264.7 cell lines have demonstrated that Panx1 enables the entry of bacterial signals such as muramyl dipeptide (MDP) into the cytoplasm, promoting inflammasome activation and IL-1β release. Specifically, Marina-García et al. (39) showed that the cytosolic relocalization of fluorescent MDP from acidified vesicles required functional Panx1 in ATP-stimulated macrophages. This finding also suggests a potential link between Panx1 and antigen presentation and cross-presentation (40) this remains an unexplored function for this channel (Figure 3). Furthermore, Kanneganti et al. (41) reported that Panx1 was essential for caspase-1 activation induced by various bacterial PAMPs, including MDP, but not by flagellin in the presence of ATP. However, flagellin did induce caspase-1 activation when directly introduced into the cytosol by pore-forming proteins, suggesting differential recognition and delivery mechanisms depending on the PAMP. Pharmacological inhibition of Panx1 significantly reduced this activation, highlighting its importance in rapid inflammatory responses against bacterial infections.

In addition to its role in initiating a proinflammatory response, Panx1 can also modulate the release of other inflammatory cytokines and chemokines in macrophages and monocytes. An *in vitro* study using human monocytes observed that Panx1 activation by Toll-like receptor 2 (TLR2) agonists induces IL-1β release (42). However, IL-1β release induced by TLR4 agonists was independent of Panx1, suggesting that Panx1’s involvement in cytokine release may depend on the specific inflammatory stimulus. The migration of macrophages and monocytes to sites of inflammation is a crucial process in the innate immune response. Some studies suggest that Panx1 may be involved in regulating the migration of these cells. In an *in vitro* study using macrophages, Panx1 inhibition was found to reduce ATP-induced chemotaxis (43). However, further research is needed to confirm the role of Panx1 in macrophage and monocyte migration *in vivo*. The involvement of Panx1 in innate immunity is supported by compelling evidence across various cell types and models. Its role in ATP release, inflammasome activation, and cytokine modulation positions it as a central mediator of early inflammatory responses. Moreover, its potential function in facilitating cytosolic entry of bacterial components opens intriguing questions about its contribution to antigen presentation and immune activation. However, the stimulus-dependent variability in Panx1 function and the incomplete understanding of its role in cell migration underscore the need for further mechanistic studies. Elucidating these context-specific roles may advance the development of targeted interventions for inflammatory and infectious diseases.

### 3.3 Panx1 in neutrophils

Studies in human neutrophils and *in vitro* chemotaxis models, using specific pharmacological inhibitors and differentiated neutrophilic HL-60 cells, revealed that Panx1 facilitates the activation of A2A adenosine receptors at the rear of the cells, leading to cAMP accumulation and inhibition of chemotactic signaling. This mechanism ensures a balance between activation and suppression signals within chemotactic gradients, which is crucial for proper cell orientation in response to inflammatory stimuli (44). Additionally, Panx1 has been implicated in acute vascular inflammation. Using mice with specific Panx1 deletion in endothelial cells and a systemic inflammation model induced by TNF-*α*, it was observed that Panx1 regulates ATP release in the venous endothelium, facilitating leukocyte adhesion and extravasation through the venous walls. This process depends on Panx1 phosphorylation mediated by Src family kinases, positioning these channels as key mediators in vascular inflammation. Moreover, the same study demonstrated that ATP released via Panx1 also activates venous endothelial cells, amplifying the inflammatory response (38).

On the other hand, Panx1 also plays a fundamental role in cardiovascular and chronic inflammatory diseases. In a murine model of abdominal aortic aneurysm induced by pancreatic elastase, it was found that Panx1 channels in platelets regulate neutrophil migration to the aortic wall through direct cell–cell interactions and endothelial cell activation. However, specific deletion of Panx1 in platelets did not affect extracellular matrix remodeling or aneurysm progression, suggesting that its impact is limited to inflammation modulation (45). In the context of non-ischemic heart failure, a murine model with cardiomyocyte-specific Panx1 deletion revealed that this protein influences glycolytic metabolism and reduces isoproterenol-induced cardiac hypertrophy. Additionally, decreased neutrophil infiltration in the myocardium was observed, highlighting the protective role of Panx1 absence in chronic cardiac inflammation (46). These findings underscore the relevance of Panx1 as a therapeutic target in inflammatory and cardiovascular diseases, given its role in modulating complex inflammatory and metabolic processes.

Neutrophil extracellular traps (NETs) are extracellular DNA structures released by neutrophils in response to various stimuli, including bacterial and fungal infections, sterile inflammation, and certain cancers (47). These structures contain DNA, histones, and other proteins that can trap and kill microorganisms but may also contribute to tissue damage and inflammation in some diseases (48).

Panx1 has been implicated in NET formation. In an *in vitro* study using bovine neutrophils, it was demonstrated that oleic and linoleic fatty acids induce NET release through a Panx1-dependent mechanism. These fatty acids activate Panx1, leading to ATP release and the activation of the purinergic receptor P2X1. P2X1 activation triggers a signaling cascade that ultimately results in NET formation (49).

**Figure 4:**
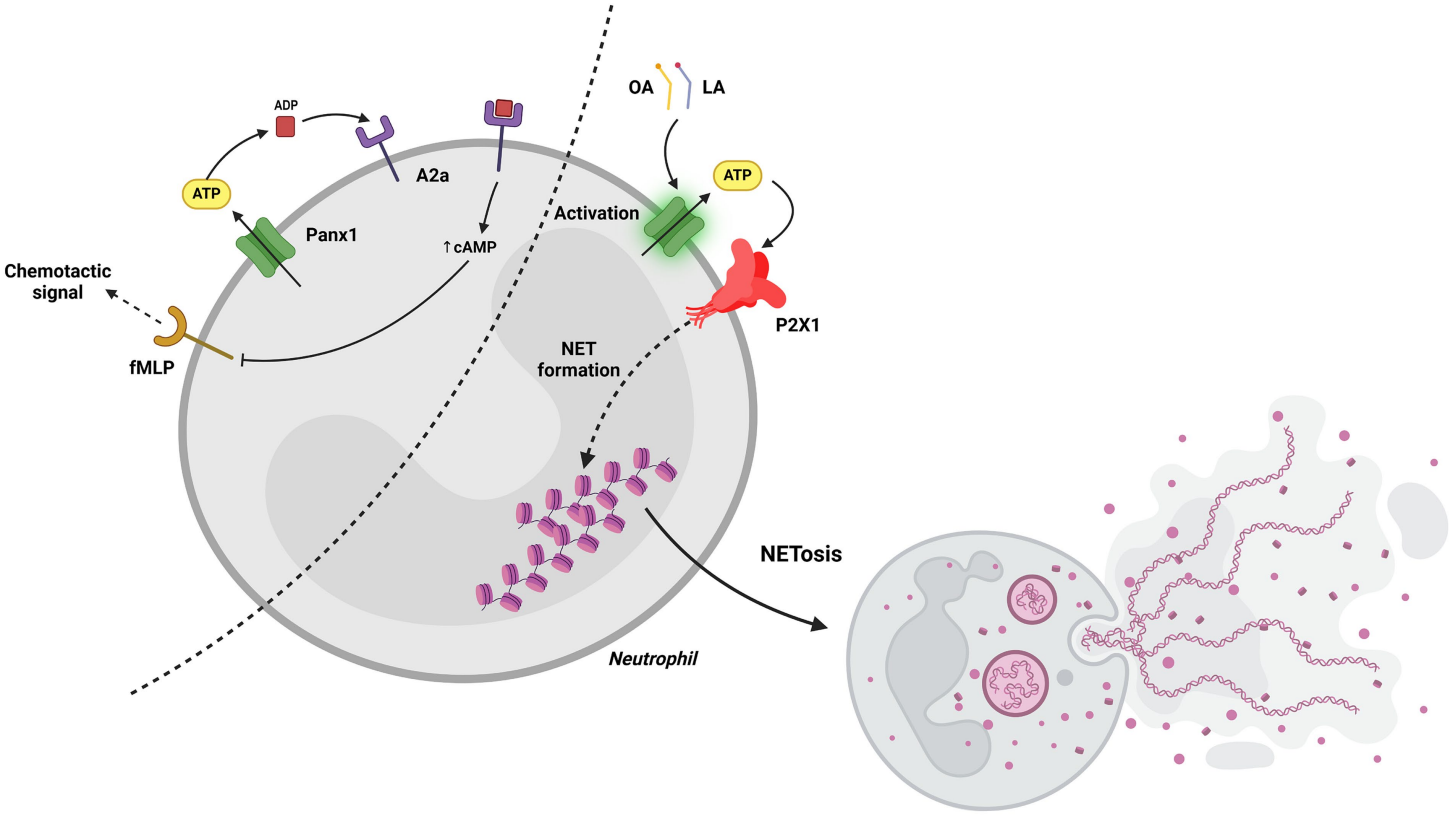
Role of Panx1 in neutrophils. On the left, the figure illustrates how Panx1 facilitates the activation of A2A adenosine receptors through ATP release into the extracellular space, leading to cAMP accumulation and inhibition of chemotactic signaling. On the right, it shows that activation with oleic or linoleic acid promotes ATP release in a Panx1-dependent manner; this ATP then activates the purinergic receptor P2X1, triggering a signaling cascade that ultimately leads to the formation of NETs. Created with BioRender.com.

In another study using neutrophils from WT and Panx1-KO mice, Panx1 was shown to facilitate ATP release, modulating NETosis in response to stimuli such as A23187 and PMA. Genetic and pharmacological inhibition of Panx1 significantly delayed and reduced NETosis. Additionally, ATP amplified this process through purinergic receptors, although it did not initiate it independently. These findings establish Panx1 as a key regulator and a potential therapeutic target in exacerbated inflammatory diseases (50) (Figure 4). Panx1 plays a pivotal role in neutrophil function, from guiding chemotaxis to promoting NET formation and vascular inflammation. These findings highlight its potential as a therapeutic target in inflammatory and cardiovascular diseases. Still, more research is needed to clarify how Panx1 balances protective and pathological responses across different contexts.

### 3.4 Pannexin 1 in dendritic cells

**Figure 5:**
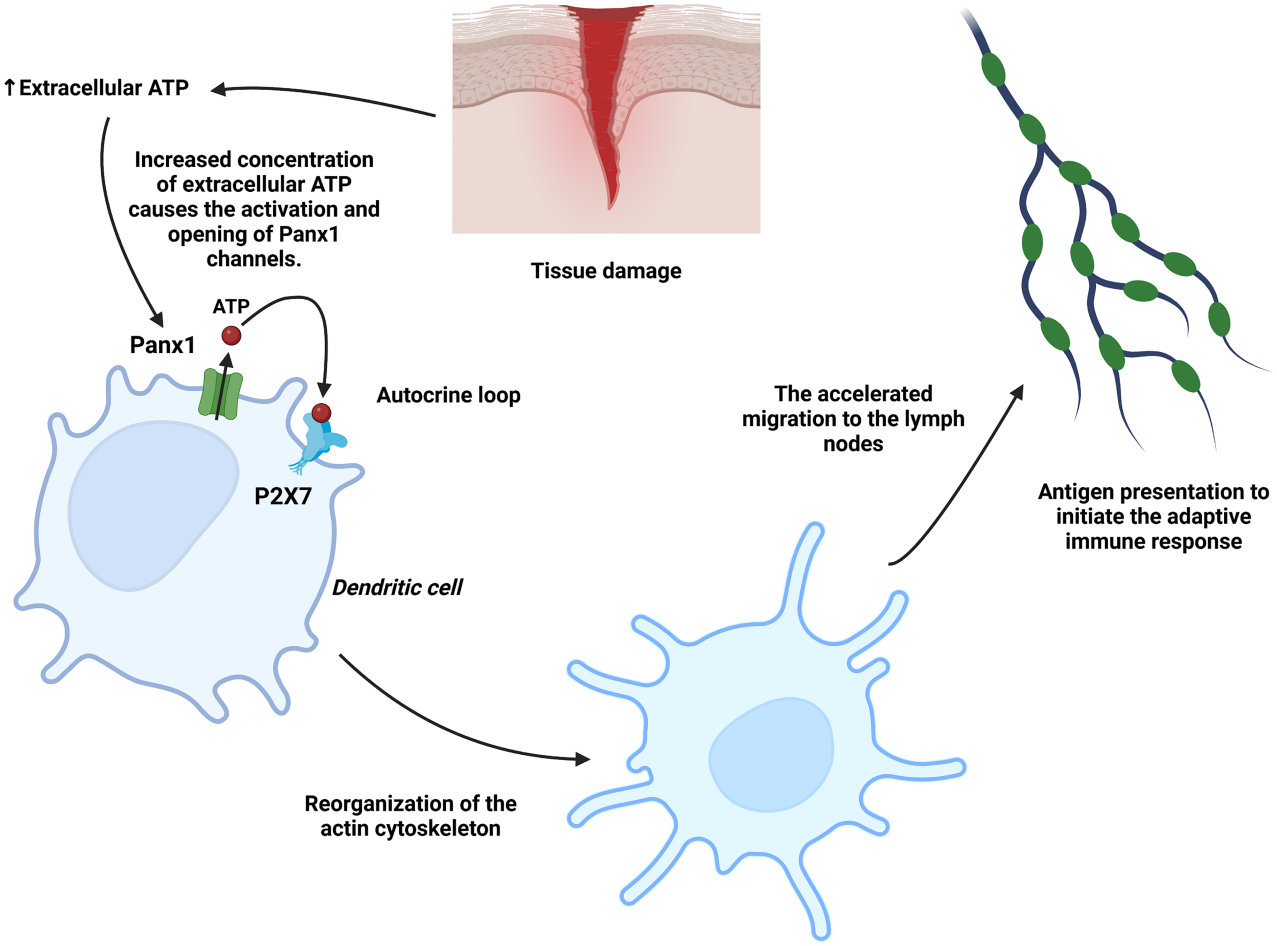
Role of Panx1 in dendritic cells. Under tissue injury conditions, ATP acts as a danger signal in dendritic cells, inducing Panx1 activation and the subsequent additional release of ATP into the extracellular space. This ATP activates the P2X7 receptor in an autocrine loop, stimulating actin cytoskeleton reorganization and promoting accelerated migration toward lymph nodes, where dendritic cells present antigens. Created with BioRender.com.

Panx1 plays a crucial role in regulating the motility and immune function of dendritic cells (DCs). This mechanism relies on ATP release through Panx1, which acts as a danger signal, promoting the activation of purinergic P2X7 receptors in DCs (51). This process stimulates actin cytoskeleton reorganization and accelerates migration toward lymph nodes, where these cells initiate the adaptive immune response (52). Experimental models, including bone marrow-derived DCs from Panx1 knockout mice and migration assays in microfabricated devices, demonstrated that the absence of Panx1 significantly reduces DC migration speed, although it does not affect the initial Ca^2+^ response mediated by P2X7. These findings highlight the importance of Panx1 in establishing an autocrine purinergic loop that amplifies motility and migration signals in response to ATP stimuli (53) (Figure 5).

**Figure 6:**
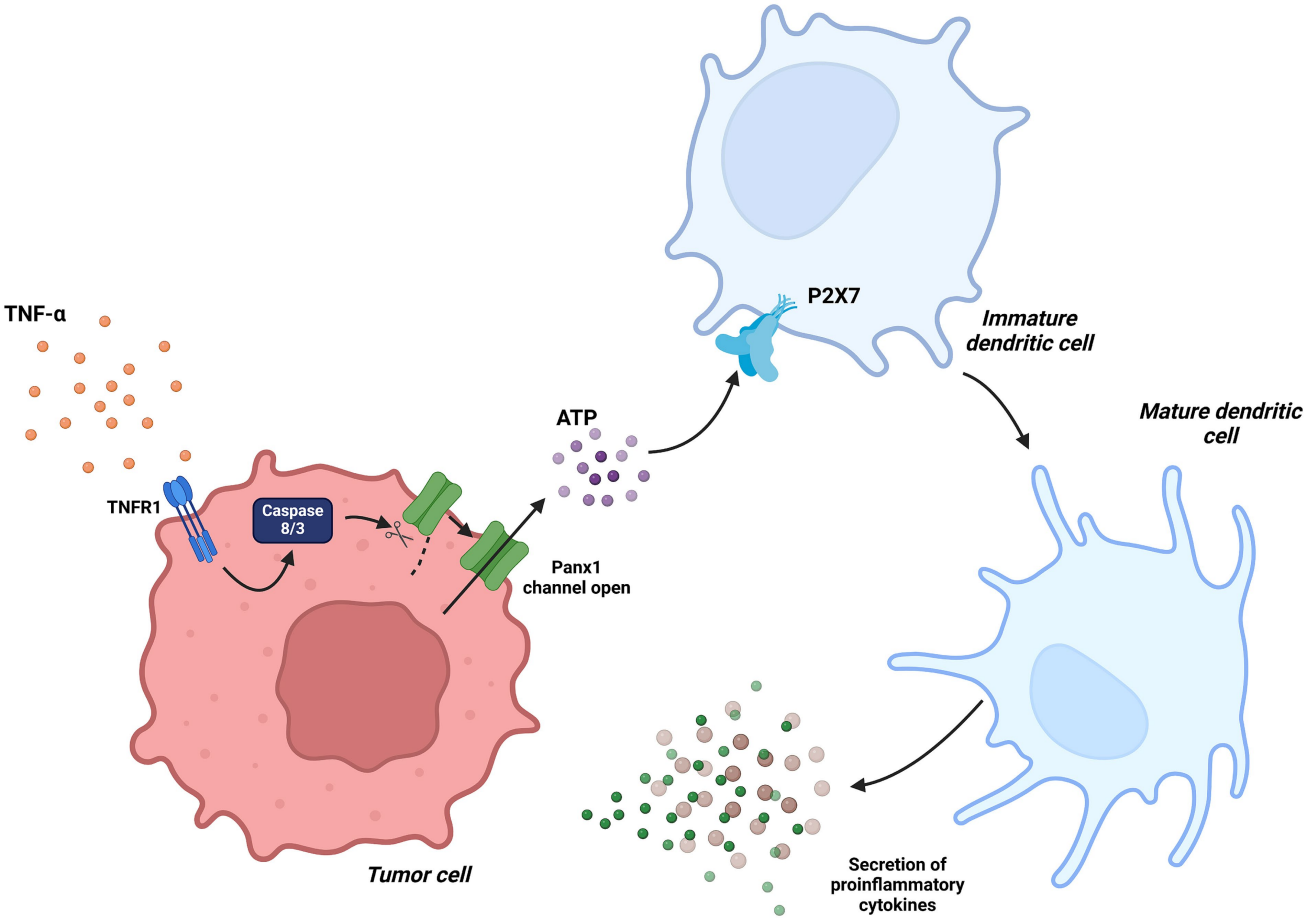
Dendritic Cells in tumor immunity associated with Panx1. In the context of chemotherapy-induced tumor immunity, TNF-*α* signaling activates caspase-3, which proteolytically processes Panx1, maintaining it in a constantly active state. This mechanism facilitates ATP release into the tumor microenvironment, promoting dendritic cell maturation through the activation of the P2X7 receptor. Created with BioRender.com.

Furthermore, in the context of chemotherapy-induced tumor immunogenicity, Panx1 facilitates ATP release through a caspase-3-dependent mechanism activated by TNF-*α* (54). This ATP activates P2X7 receptors within the tumor microenvironment, promoting DC maturation, proinflammatory cytokine secretion, and the infiltration of cytotoxic T cells. In murine models of colorectal cancer, Panx1 inhibition resulted in a significant reduction in the immune response induced by chemotherapy, whereas the administration of an ATP analog enhanced therapeutic efficacy. These findings underscore the essential role of the Panx1-ATP-P2X7 axis in regulating DC functions under pathological conditions such as cancer and chronic inflammation (33) (Figure 6).

### 3.5 Pannexin 1 in T cell activation and differentiation

Woehrle et al. (55) reported the role of pannexin-1 hemichannels and P2X1 and P2X4 receptors in T cell activation at the immune synapse. Their findings indicate that T cell receptor (TCR) stimulation induces the translocation of these components to the synapse, facilitating ATP release and calcium (Ca^2+^) influx, both of which are essential for T cell activation in a Jurkat cell model. Inhibition of these receptors or pannexin-1 significantly reduced Ca^2+^ entry and IL-2 expression, a key activation marker.

Other studies, such as Vardam-Kaur et al. (25), revealed that Panx1 is essential for effector and memory responses of CD8+ T cells against viral infections (*LCMV-Armstrong virus*) and tumors (*B16-gp33 melanoma*). Using murine models with T cell-specific Panx1 deletion and adoptive transfer experiments, it was demonstrated that Panx1 facilitates initial activation through ATP export and P2RX4 receptor activation, while it promotes effector differentiation via extracellular lactate accumulation. At the memory stage, Panx1 supports cell survival through P2RX7 receptor activation, enhancing mitochondrial respiration and AMPK pathway activation. Panx1 deficiency reduced CD8+ T cell proliferation and impaired their ability to control tumors and respond to infections (25).

A study demonstrated that hypersaline stress regulates T cell function through pannexin-1 (Panx1) hemichannels and purinergic receptors P2X1, P2X4, and P2X7. Using Jurkat T cells and *in vitro* osmotic stress models, it was observed that treatment with hypertonic saline (HS) solution induces ATP release mediated by Panx1, which activates p38 MAPK signaling and increases IL-2 transcription, a key factor in T cell activation. Inhibition of Panx1 or P2X receptors blocked these effects (56). Medina et al. (57) demonstrated that Pannexin-1 channels regulate communication between regulatory T cells (Treg) and effector T cells (Teff) to limit allergic airway inflammation. Using murine models with global and T cell-specific deletion, the researchers identified that Panx1 facilitates ATP release in immune microenvironments, promoting its conversion into immunosuppressive adenosine via CD39 and CD73, which is crucial for Teff suppression by Tregs. In a model of dust mite-induced inflammation, Panx1 deficiency exacerbated pulmonary inflammation, while its reexpression in T cells reduced pathology.

**Figure 7:**
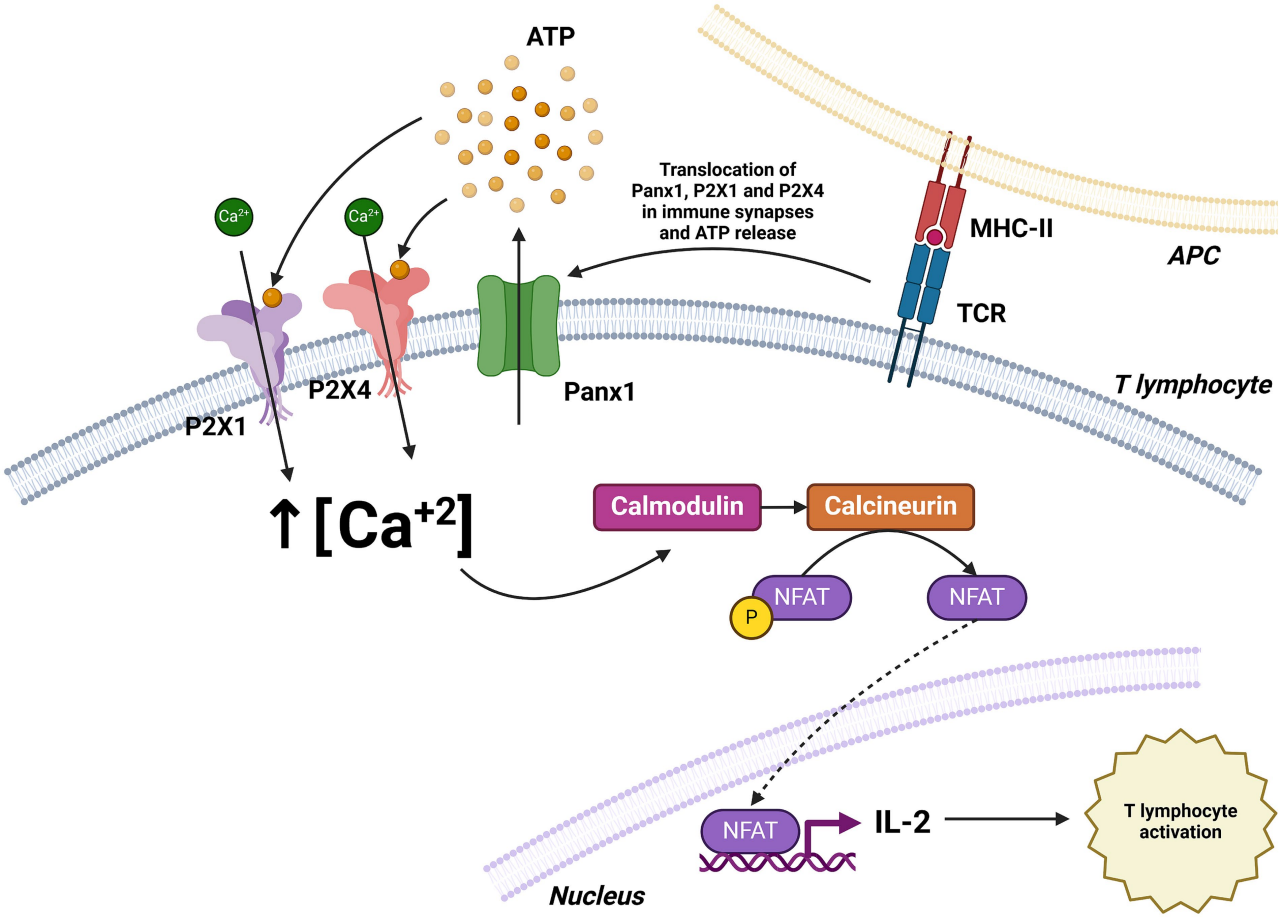
Role of Panx1 in T cell activation. T cell receptor (TCR) stimulation induces the translocation of Panx1, P2X1, and P2X4 to the immune synapse, facilitating ATP release and Ca^2+^ influx. The increase in intracellular Ca^2+^ concentration activates the calmodulin pathway, leading to enhanced IL-2 expression and T cell activation. Created with BioRender.com.

In line with previous findings, a recent report showed that Panx1 influences the activation and memory formation of CD8+ T cells. Using a murine model with T cell-specific Panx1 deletion, researchers observed a decrease in the frequency of effector and memory CD8+ T cells following viral infections. Additionally, Panx1 deficiency resulted in reduced T cell receptor signaling, lower proliferation and cytokine production, and increased cell apoptosis (58) (Figure 7).

## 4 Pannexin 1 in disease contexts

### 4.1 Pannexin 1 and tumor progression

Pannexin 1 plays key roles in multiple aspects of tumor development, including cell migration, metabolism, and immune response within the tumor microenvironment. This channel regulates the release of ATP and other signaling molecules that influence cell proliferation, migration, and invasion. In breast cancer, Panx1 has been shown to be highly expressed in metastatic foci and is associated with an enhanced epithelial-mesenchymal transition (EMT). *In vitro* models using breast cancer cell lines such as MDA-MB-231 and MCF-7, as well as transcriptomic data analyses from patient samples, revealed that pharmacological or genetic inhibition of Panx1 reverses the EMT phenotype and reduces cellular invasiveness. In the context of tumor progression, Panx1 plays key roles in multiple aspects, including cell migration, metabolism, and the immune response within the tumor microenvironment. In particular, high Panx1 expression in basal-like breast cancer has been shown not only to be associated with enhanced epithelial-mesenchymal transition (EMT) but also to lead to neutrophil recruitment and the formation of a high adenosine immunosuppressive tumor microenvironment. Furthermore, studies in melanoma cells have identified that Panx1 deletion increases T lymphocyte infiltration within the tumor microenvironment, suggesting a dual role in tumor and immune regulation (59, 60).

In the context of cell migration and survival, Panx1 has also been shown to be crucial in the metastasis of various cancers. A study in melanoma cells identified that Panx1 directly interacts with *β*-catenin, regulating the Wnt signaling pathway and promoting tumor growth and immune evasion. *In vitro* melanoma models and transgenic BRAF/Pten mouse models demonstrated that Panx1 deletion increases T lymphocyte infiltration within the tumor microenvironment, although it does not significantly affect primary tumor formation, suggesting a dual role in tumor and immune regulation (61, 62). Additionally, in high-risk neuroblastoma, inhibition of Panx1 channels using carbenoxolone and probenecid halted cell proliferation and reduced tumor growth, highlighting the therapeutic potential of Panx1 blockade in this pediatric cancer (63).

**Figure 8:**
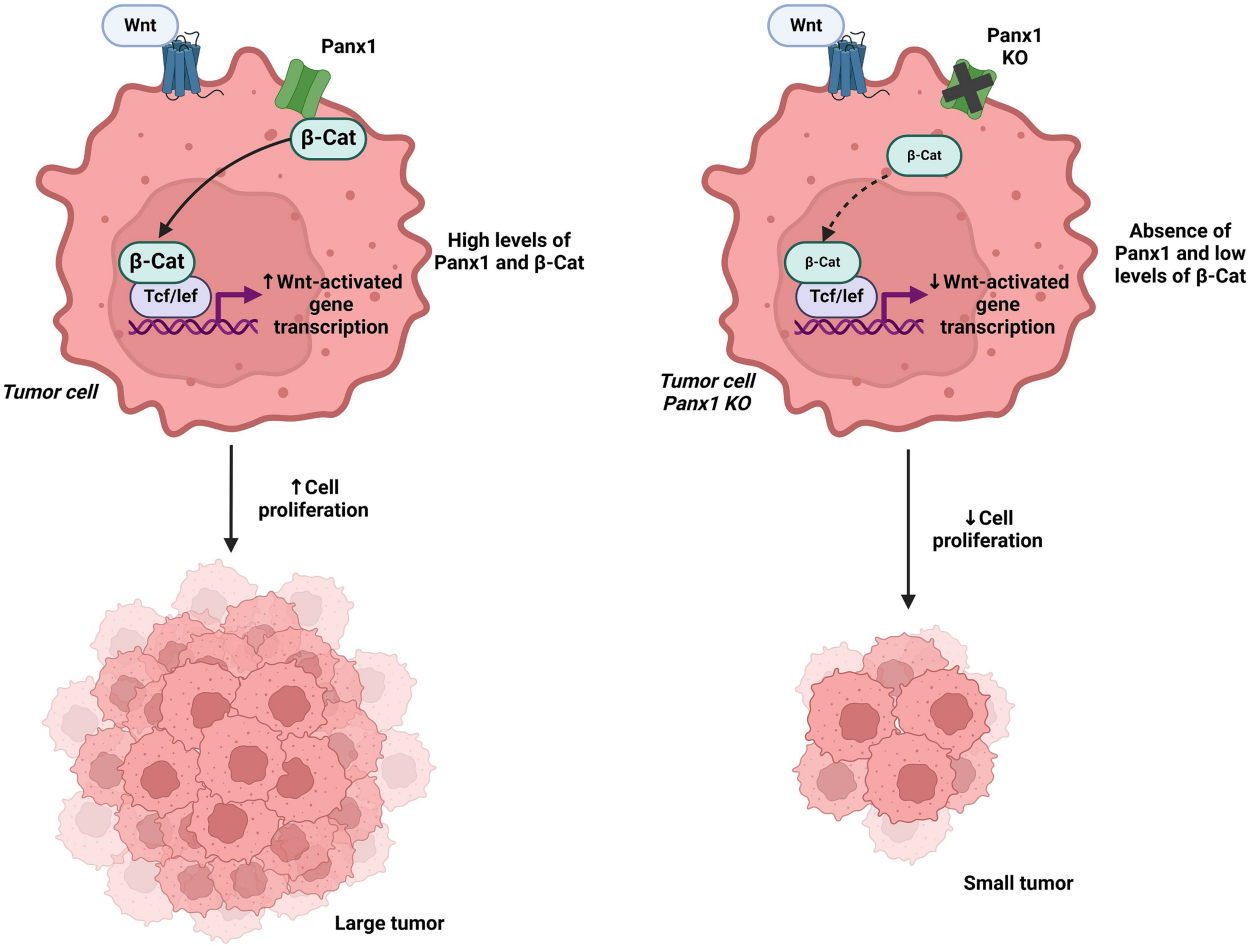
Interaction of Panx1 with β-catenin and its impact on Wnt signaling. In the left panel, Panx1 is shown to directly interact with β-catenin, regulating the Wnt signaling pathway and promoting tumor growth. In the right panel, Panx1 KO tumor cells exhibit reduced Wnt pathway activation, leading to decreased cell proliferation. Created with BioRender.com.

**Figure 9:**
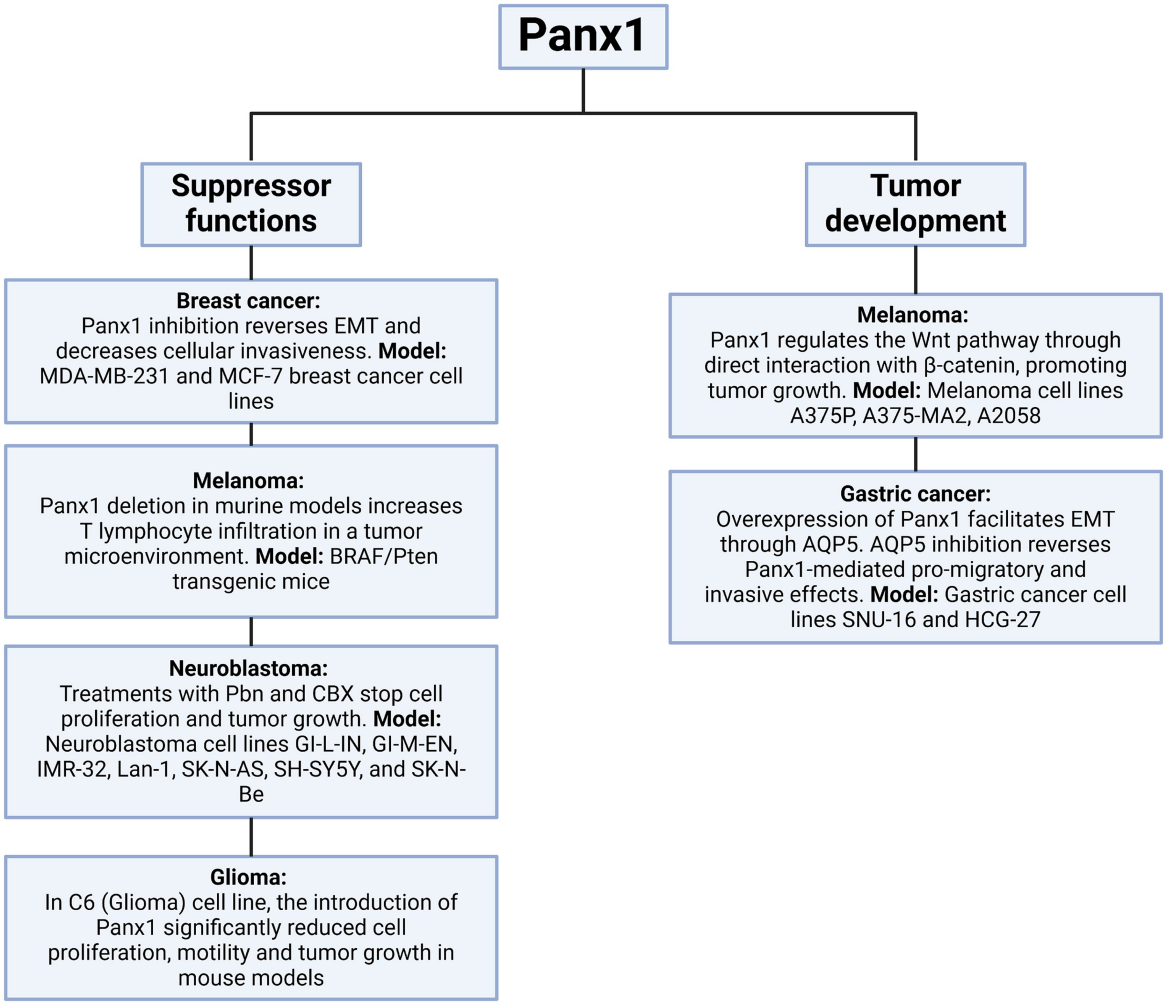
Dual role of Panx1 in tumor progression. This diagram illustrates the role of Panx1 in the tumor context. On the left, its function as a tumor suppressor is depicted, observed when its activity is inhibited, or its expression is altered in various experimental models. On the right, the pathways through which Panx1 can promote tumor development are detailed, including enhanced proliferation, migration, and cell invasion. Created with BioRender.com.

The impact of Panx1 also extends to gastric cancer, where its overexpression facilitates epithelial-mesenchymal transition (EMT) through the regulation of aquaporin 5 (AQP5), a critical mediator of signaling pathways involved in cell migration and invasion. *In vitro* models using gastric cancer cell lines revealed that AQP5 inhibition reverses the pro-migratory and invasive effects mediated by Panx1, further establishing the functional connection between these molecules in gastric cancer progression (64). In contrast to the tumor-promoting roles discussed earlier, some studies have suggested a potential tumor-suppressive function of Panx1 in specific contexts. For instance, in rhabdomyosarcoma, Panx1 re-expression not only decreased cell viability, inhibited migration, and promoted apoptosis, but also exhibited an inhibitory interaction with AHNAK, highlighting its role as a regulator of malignancy in this pediatric sarcoma (65). Similarly, in C6 glioma cells, Panx1 reintroduction significantly reduced cell proliferation, motility, and tumor growth in mouse models, indicating that its function may vary depending on cancer type and the surrounding microenvironment (52). This duality in Panx1 function could be influenced by tumor stage, molecular context, or interactions with specific proteins, such as AHNAK in rhabdomyosarcoma or *β*-catenin in melanoma (62, 65) (Figures 8, 9). Panx1 exerts complex, context-dependent roles in cancer, acting as both a promoter and suppressor of tumor progression depending on the tumor type and microenvironment. Its influence on EMT, immune evasion, and cell migration underscores its relevance as a potential therapeutic target. However, its dual behavior highlights the need for precise characterization before clinical application.

### 4.2 Pannexin 1 and immune response in SARS-CoV-2 infection

Panx1 has emerged as a key player in the pathogenesis of COVID-19, primarily by mediating ATP release into the extracellular space. This extracellular ATP functions as a danger-associated molecular pattern (DAMP), activating purinergic receptors such as P2X7 and P2Y2 and promoting downstream inflammatory responses. In the context of SARS-CoV-2 infection, the viral Spike S1 protein has been shown to induce sustained opening of Panx1 channels in human pulmonary epithelial cells, triggering the release of ATP, prostaglandin E2 (PGE2), and interleukin-1β (IL-1β). These events depend on interactions with the ACE2 receptor, the furin protease, and endocytosis, highlighting the role of Panx1 in exacerbating pulmonary inflammation and severe respiratory dysfunction observed in COVID-19 (26).

The impact of Panx1 channels is not limited to pulmonary damage but also extends to systemic inflammation and the cytokine storm characteristic of severe COVID-19 cases. Preclinical models have identified that Panx1 activation contributes to immune cell recruitment and amplifies inflammatory signaling through its interaction with purinergic receptors. Furthermore, inhibition of these channels with drugs such as probenecid has been shown to significantly reduce inflammation and viral replication, suggesting a promising therapeutic approach for managing the disease (66–68). For instance, in experimental models, pharmacological inhibition of Panx1 decreased extracellular ATP levels, a key molecule in exacerbated inflammatory signaling, improving survival under viral infection conditions (26, 66).

Beyond its role in inflammation, Panx1 also influences pathogenesis at the cellular and molecular levels. Recent studies have described that SARS-CoV-2-induced Panx1 activation promotes the activation of the NLRP3 inflammasome, a protein complex that drives the production of proinflammatory cytokines such as IL-1β and IL-18, exacerbating tissue inflammation. Additionally, high levels of ATP released through Panx1 have been documented to promote cell apoptosis and endothelial damage, contributing to systemic complications of COVID-19, including coagulopathy and acute respiratory distress syndrome (66, 69, 70).

**Figure 10:**
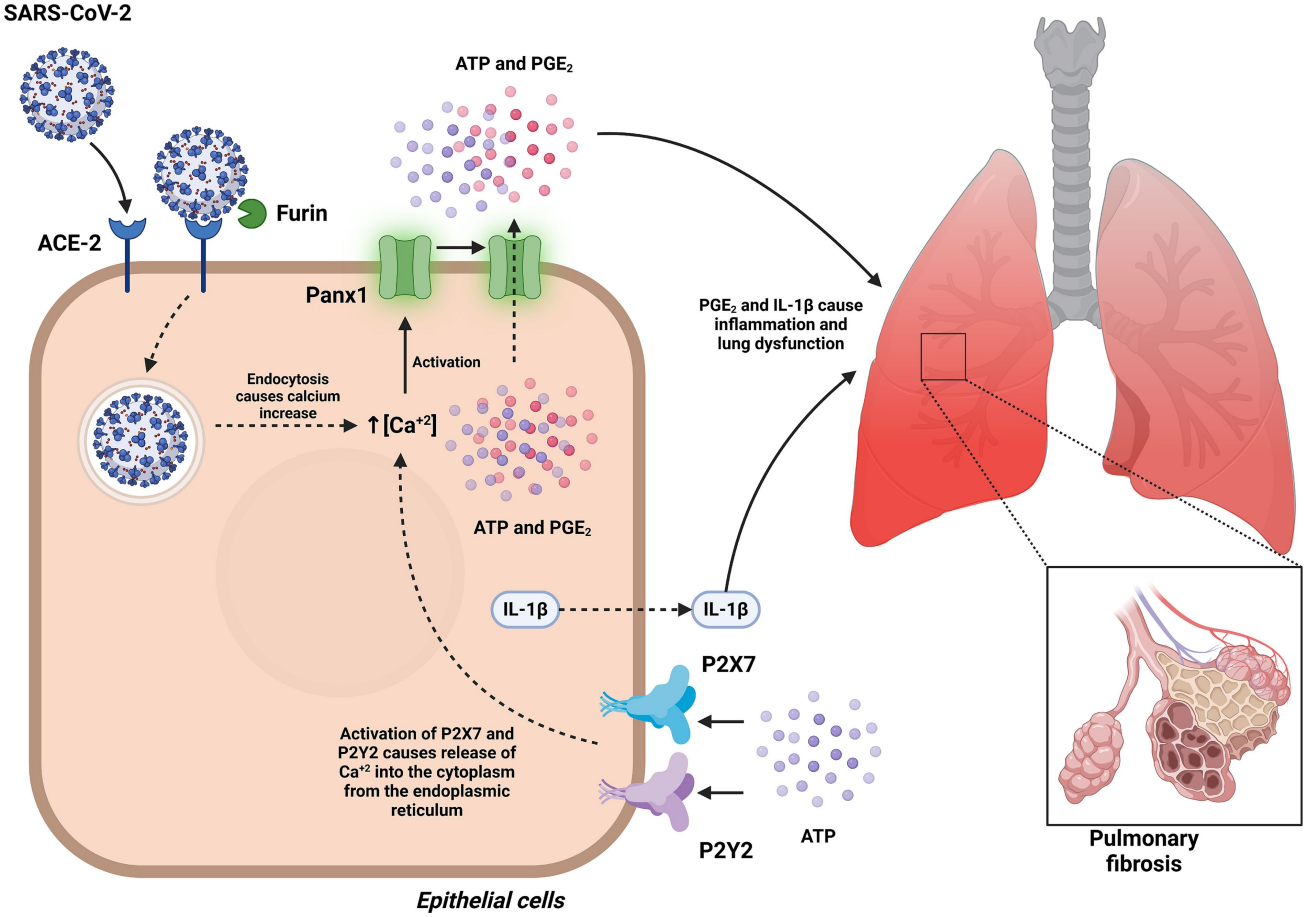
Role of Panx1 in the pathogenesis of COVID-19. This figure illustrates how Panx1 mediates inflammation and regulates exacerbated immune responses during SARS-CoV-2 infection. In pulmonary epithelial cells, the interaction of the Spike S1 protein with the ACE2 receptor induces sustained opening of Panx1 channels in a furin-dependent manner, facilitating ATP release. ATP acts as a danger signal by activating purinergic receptors P2X7 and P2Y2, triggering the release of PGE2 and IL-1β. This process contributes to the exacerbation of pulmonary inflammation and severe respiratory dysfunction observed in COVID-19. Created with BioRender.com.

Drugs that were investigated as potential COVID-19 treatments, such as hydroxychloroquine and favipiravir, have been shown to inhibit Panx1 activity without affecting its gene or protein expression, highlighting their potential to modulate inflammation without interfering with other physiological functions of Panx1 (67). It is important to note that despite initial investigations, extensive clinical trials have not demonstrated a credible benefit of hydroxychloroquine for COVID-19 treatment (71). These findings emphasize the importance of further investigating Panx1 not only as a severity biomarker but also as a therapeutic target in the management of COVID-19 and its associated complications (Figure 10).

### 4.3 Pannexin 1 as a therapeutic or pharmacological target in inflammatory and autoimmune diseases

Panx1 has emerged as a promising therapeutic target in a wide range of inflammatory and autoimmune diseases due to its role in extracellular ATP release and the activation of inflammatory pathways. This channel is associated with the amplification of inflammation through the activation of purinergic receptors such as P2X7, which regulate the release of proinflammatory cytokines, immune cell recruitment, and inflammasome formation. For instance, in experimental models of autoimmune encephalomyelitis, Panx1 inhibition with compounds such as carbenoxolone has been shown to significantly reduce disease progression and neuroimmune inflammation (72, 73). This mechanism is also evident in sepsis models, where Panx1 blockade with probenecid decreases serum ATP levels, reduces IL-1β release, and improves cellular energy metabolism, ultimately minimizing tissue damage (74, 75).

In the context of cardiovascular diseases, Panx1 has also been identified as a key mediator in inflammatory processes associated with endothelial damage. In myocardial infarction models, Panx1 inhibition attenuates myocardial inflammation by reducing ATP release and inflammasome activation in endothelial cells, thereby promoting better functional recovery of the affected tissue (76). Additionally, in chronic vascular inflammation, Panx1 regulates leukocyte trafficking and endothelial cell activation, making it a viable pharmacological target for mitigating persistent inflammatory pathologies (77, 78).

The relevance of Panx1 also extends to autoimmune disorders such as multiple sclerosis and rheumatoid arthritis. Studies in animal models have demonstrated that Panx1 inhibition reduces T lymphocyte infiltration into the central nervous system and improves motor deficits associated with these diseases (79, 80). These findings are consistent with previous observations that Panx1 modulates T cell function, particularly by facilitating ATP release and adenosine production at the Treg–Teff interface, thereby influencing immune regulation. In addition, Panx1 regulates the osteogenic differentiation of mesenchymal stem cells through p38 MAPK phosphorylation, highlighting its broader role in autoimmune diseases affecting bone tissue (81).

**Figure 11:**
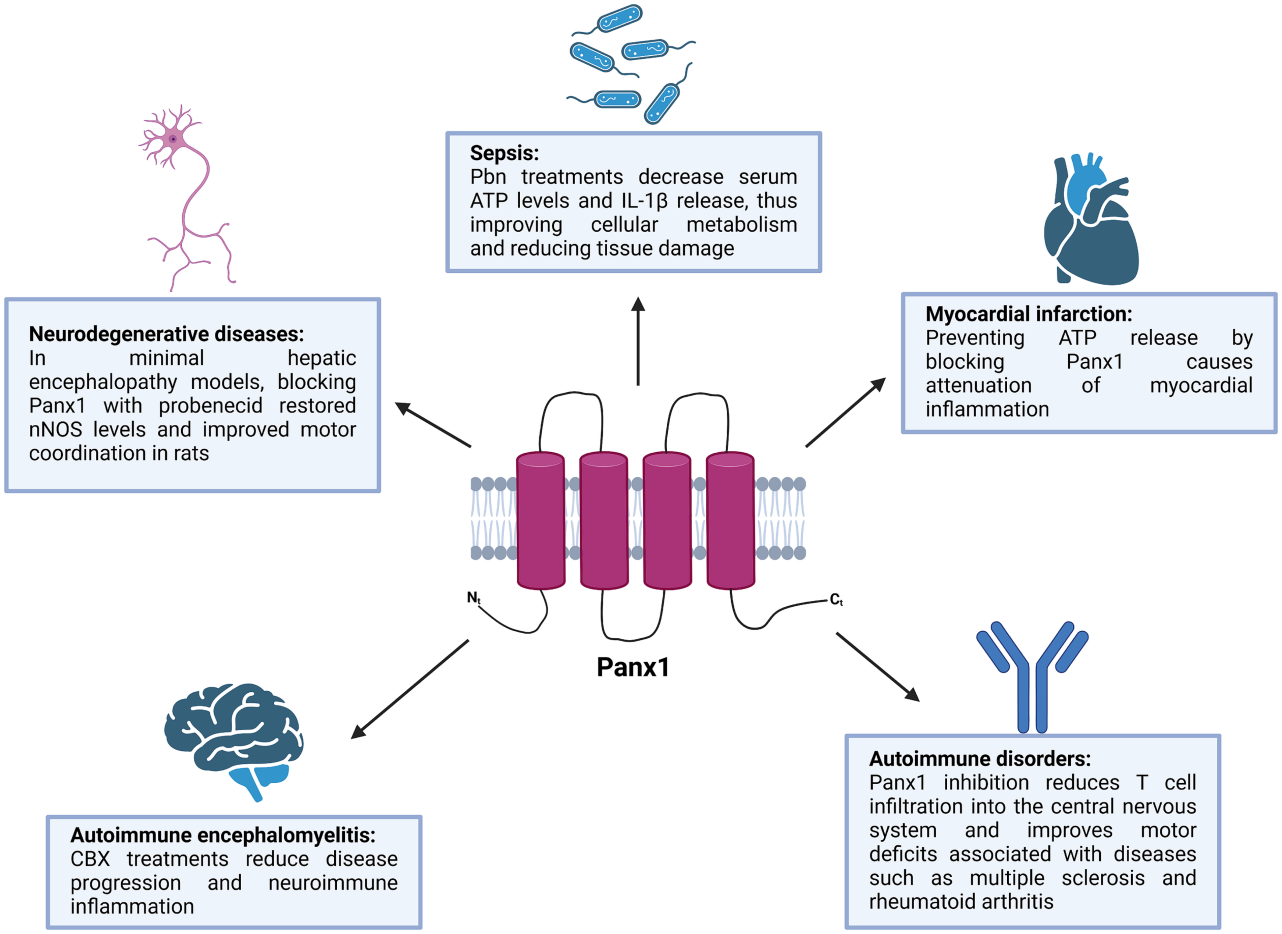
Therapeutic use of Panx1 inhibitors in inflammatory diseases. This figure represents the role of Panx1 in various diseases associated with inflammatory processes. It highlights the inhibitory drugs that have been shown to attenuate disease progression and clinical symptoms, emphasizing their therapeutic potential in inflammatory, autoimmune, and neurodegenerative conditions. Created with BioRender.com.

**Figure 12:**
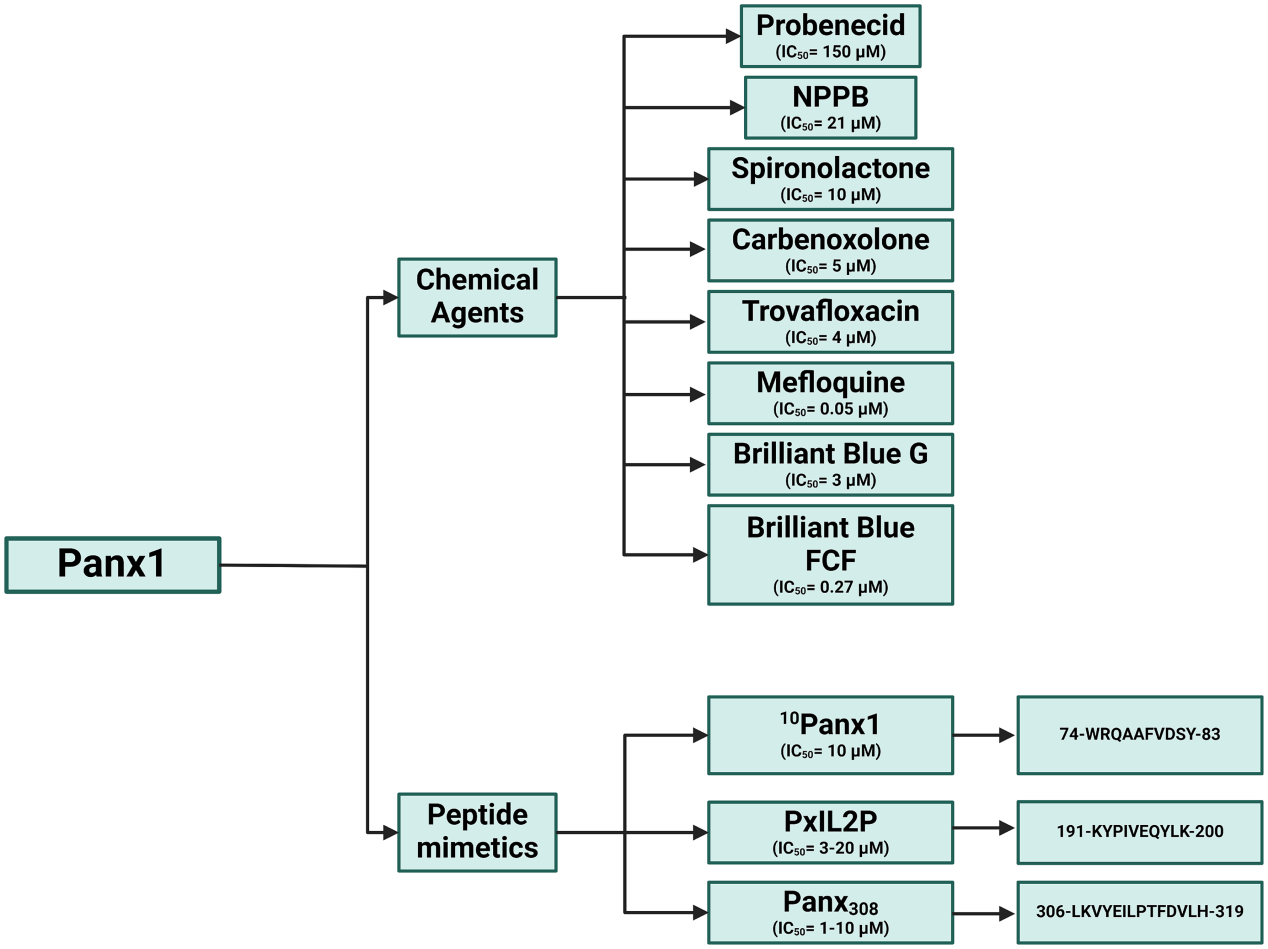
Panx1 inhibitors: Chemical compounds and mimetic peptides. This diagram illustrates various inhibitors targeting Panx1, including chemical compounds and mimetic peptides. It highlights the mechanisms of action of each inhibitor and their potential to modulate channel function in different physiological and pathological contexts. Created with BioRender.com.

In neurodegenerative diseases such as stroke and hepatic encephalopathy, Panx1 inhibition has shown promising results in mitigating neuronal damage and improving cognitive function. For instance, in models of minimal hepatic encephalopathy, Panx1 blockade with probenecid restored nNOS levels and improved motor coordination in rats (82). Similarly, in subarachnoid hemorrhage, Panx1 inhibition reduced AIM2 inflammasome activation and early neuronal damage, highlighting its role in neuroprotection (83). Current studies indicate that Panx1 modulation may offer significant therapeutic benefits across a wide range of inflammatory and autoimmune diseases (Figure 11). By acting as a key mediator of inflammation and tissue damage, Panx1 emerges as a versatile pharmacological target with potential applications in treating conditions involving chronic inflammation and immune dysfunction (84, 85). To date, various chemical inhibitors and peptides have been described for studying Panx1, advancing the understanding of its biological function. However, further research is essential to elucidate its specific mechanisms of action and develop more selective inhibitors. This would not only help minimize side effects but also maximize clinical efficacy, complementing and expanding current knowledge in this field (Figure 12). To date, and according to the literature reviewed in this manuscript, Panx1 inhibitors and modulators have demonstrated considerable therapeutic potential in numerous preclinical models of inflammatory, autoimmune, cardiovascular, and neurodegenerative diseases. However, it is important to note that, as of this review, there is no published evidence indicating that compounds specifically developed to modulate Panx1 have advanced to human clinical trials for these or other conditions (86). It is important to distinguish between existing drugs that incidentally inhibit Panx1 and compounds whose primary mechanism and therapeutic target is Panx1. Drugs such as probenecid (used for gout) or spironolactone (for hypertension) have been shown to inhibit Panx1 in laboratory studies. However, this article highlights that the concentrations required for significant inhibition of Panx1 by probenecid are very high, and its lack of specificity “undermines its potential for therapeutic use in humans” as a Panx1 inhibitor. Similarly, although spironolactone inhibits Panx1, its main clinical action is attributed to mineralocorticoid receptor antagonism, and the contribution of Panx1 inhibition to its therapeutic effects remains under investigation. Other Panx1 inhibitors that have been identified, such as trovafloxacin, have been ruled out for therapeutic use due to severe side effects. The context-dependent effects of Pannexin 1 are summarized in Table 1, highlighting its dual role as both a pro-inflammatory and anti-inflammatory mediator. These contrasting outcomes are shaped by factors such as the specific cell type, the nature of the inflammatory stimulus, and interactions with key signaling molecules like P2X7 receptors and caspases. This duality underscores the complexity of Panx1 signaling and its potential as a selective therapeutic target in diverse pathological conditions.

**Table 1:** Context-dependent roles of Panx1 in regulating inflammation.

Panx1 role	Mechanism/effect	Key factors (cell type, stimulus, interacting proteins)	References
Pro-inflammatory	ATP release & amplification of purinergic signaling	General	Penuela et al. (3), Ransford et al. (4), and Chen et al. (5)
	Inflammasome activation (NLRP3) and IL-1β/IL-18 release	Macrophages, Monocytes; ATP, P2X7 receptor, bacterial signals (MDP)	Silverman et al. (20), Pelegrin and Surprenant (32), Marina-García et al. (39) and Kanneganti et al. (41)
	Release of inflammatory cytokines (IL-1β, TNF-α, IL-6) and chemokines (CCL2, CXCL1)	Various cells (e.g., colorectal cancer cells, brain); TNF-α, TLR2 agonists	Huang et al. (33), Seo et al. (34), and Parzych et al. (42)
	Leukocyte adhesion and migration (acute vascular inflammation)	Endothelial cells; TNF-α, Src family kinases (SFK), platelets (in aneurysm)	Lohman et al. (38) and Metz et al. (45)
	Neutrophil extracellular trap (NET) formation	Neutrophils; Oleic/linoleic fatty acids, P2X1 receptor, ATP (amplifies)	Alarcón et al. (49) and Sofoluwe et al. (50)
	Dendritic cell (DC) migration and maturation, T-cell infiltration	Dendritic cells, Tumor Microenvironment; ATP, P2X7 receptor, Caspase-3, TNF-α	Vénéreau et al. (51), Sáez et al. (53), Narahari et al. (54), and Huang et al. (33)
	T-cell activation, proliferation, effector and memory responses	T-cells (Jurkat T, CD8+ T); TCR stimulation, Ca^2+^ influx, P2X1, P2X4, P2X7 receptors, extracellular lactate, hypertonic stress, p38 MAPK	Woehrle et al. (55, 56) and Vardam-Kaur et al. (25)
	Exacerbation of inflammation in SARS-CoV-2 infection	Pulmonary epithelial cells; Viral Spike S1 protein, ACE2 receptor, furin protease, P2X7, P2Y2 receptors	Luu et al. (26), Nadeali et al. (66), Caufriez et al. (67), and Swayne et al. (68)
Anti-inflammatory (or Limiting Inflammation)	Limits pro-inflammatory cytokine release (e.g., IL-8)	HT-29 cells (necroptosis model); MLKL oligomers, RAB27A/B GTPases	Douanne et al. (35)
	Inhibition of chemotactic signaling (balancing cell orientation)	Neutrophils; ATP release, A2A adenosine receptors, cAMP accumulation	Bao et al. (44)
	Facilitates conversion of ATP to immunosuppressive adenosine, suppressing effector T cells	Regulatory T cells (Tregs); ATP, CD39, CD73	Medina et al. (57)
	Protective role in chronic cardiac inflammation (Panx1 absence reduces neutrophil infiltration)	Cardiomyocytes; isoproterenol-induced cardiac hypertrophy	Pavelec et al. (46)

## 5 Conclusion

Pannexin 1 (Panx1) has emerged as a key regulator in a wide array of physiological and pathological processes, including inflammation, cell death, immune activation, and tumor progression. The growing body of evidence highlights its remarkable functional versatility, acting not only as a conduit for ATP release but also as a signaling hub in various cellular contexts. Notably, Panx1 can exert either pro- or anti-inflammatory effects depending on the cell type, the nature of the stimulus, and its interactions with molecules such as P2X7 receptors and caspases. This dual behavior, observed in models of autoimmunity, infection, neuroinflammation, and cancer, underscores the complexity of its biological role and the importance of context when considering Panx1 as a therapeutic target.

Despite encouraging results from several preclinical studies showing that pharmacological inhibition of Panx1 can alleviate disease severity in inflammatory, autoimmune, cardiovascular, and neurodegenerative models, no Panx1-specific compound has yet advanced to clinical trials. Many of the inhibitors used thus far were not initially designed to target Panx1, and their off-target effects and limited specificity remain significant obstacles. Therefore, there is a pressing need to develop novel, selective, and potent Panx1 modulators with optimal pharmacokinetic properties that can move forward in the drug development pipeline.

In parallel, a deeper understanding of Panx1 biology is needed to identify the conditions under which its modulation would be most beneficial. This includes elucidating its cell- and tissue-specific functions, particularly within specialized immune populations, its interplay with central inflammatory pathways such as the inflammasome and necroptosis, and its contribution to the immunosuppressive tumor microenvironment through mechanisms like adenosine accumulation and immune cell recruitment. The identification of reliable biomarkers of Panx1 activity could further aid in selecting patient populations most likely to benefit from Panx1-targeted therapies.

Panx1 occupies a central position at the interface of immunity, inflammation, and disease progression. Its therapeutic potential is evident, but realizing it will require interdisciplinary efforts bridging basic science, pharmacology, and clinical research. With continued exploration, Panx1 may well transition from a compelling biological target to a concrete therapeutic strategy in the management of complex human diseases.
